# Supplementary material for: Recognising Sepsis as a Health Priority in Sub-Saharan African Country: Learning Lessons from Engagement with Gabon’s Health Policy Stakeholders
Source: Healthcare (Basel). 2022 May 10;10(5):877. doi: 10.3390/healthcare10050877 (PMC9141529; doi:10.3390/healthcare10050877)
Supplement: Supplementary file 1 [file healthcare-10-00877-s001.zip › healthcare-1646949-supplementary.pdf]

healthcare MDPI

Supplementary file

Questionnaire

i. Participant information

Please provide your information

- Stakeholder group (Please tick):

☐Ministry of Health

☐ Referral Hospital

☐ Media

☐ Research Institution

☐ ethics/regulatory institution

☐ International funder institution

- Age group (Please tick):
  ☐ 18 – 25 ☐ 26 – 30 ☐ 31 – 40 ☐ 41 +

- Gender: ☐ Male ☐ Female
- Highest qualification: ☐ Certificate/ Diploma ☐ Bachelors degree ☐ Masters degree ☐ Doctorate degree

ii. Bellwether Interview Tool

1. Currently, what three issues do you think are at the top of the [state/institutional/public] policy agenda?

2. How familiar are you with sepsis?

3. Who is involved in implementing sepsis-related policies and service delivery? Do they have the skills, relationships and incentives to deliver?

4. What individuals, constituencies or groups do you see as the main advocates for sepsis? Who do you see as the main opponents? What language are they using? What kind of evidence will convince them?

5. Considering the current educational, social and political context, do you think sepsis should be adopted as a priority disease now or in the near future? Why/why not?

6. Looking ahead, how likely do you think it is that sepsis will be adopted as a priority disease in the next five years? Why/why not? What new legislation, budgets, programmes or strategies would need to be developed that can affect sepsis?

7. If sepsis is adopted as a priority disease and an indicator of quality of the health system, what issues do you think the state needs to be most concerned about related to its prevention and management?

iii. Outcome Mapping

This brief questionnaire is part of the policy and stakeholder engagement work for the ARCS Project. For each stakeholder group, please rate the likelihood of each of its corresponding 3 outcomes being realised in the next 5 years. Your responses will remain anonymous.

<table>
<tr><th>Stakeholder Group</th><th>Questions to Consider</th><th colspan="3">Likelihood of Outcomes
(1 = Highly Unlikely; 3 = Neither Likely nor Unlikely; 5 = Highly Likely)</th></tr>
<tr><td rowspan="4">i. MOH</td><td rowspan="4">What are the influential MOH policy makers saying on sepsis? What language are they using?

How interested and open are MOH policy makers to sepsis? What kind of evidence will convince them?</td><td>Demand evidence on sepsis</td><td>Organise training workshops on sepsis for health workers</td><td>Put sepsis as the indicator of quality of health system</td></tr>
<tr><td></td><td></td><td></td></tr>
<tr><td></td><td></td><td></td></tr>
<tr><td></td><td></td><td></td></tr>
<tr><td rowspan="2">ii. Referral hospitals</td><td rowspan="2">Who is involved in implementing sepsis-related policies among central hospitals? Do they have the skills, relationships and incentives to deliver?

Are different central hospital actors working coherently</td><td>Participate in training workshops on sepsis</td><td>Accurately diagnose and report sepsis</td><td>Recognition of sepsis as a priority disease</td></tr>
<tr><td></td><td></td><td></td></tr>
</table>

**Stakeholder Group**	**Questions to Consider**	**Likelihood of Outcomes (1 = Highly Unlikely; 3 = Neither Likely nor Unlikely; 5 = Highly Likely)**		
	together to implement sepsis-related policy? Are the necessary structures and incentives in place to facilitate this?			
iii. Research institutions	How interested and open are research institutions to sepsis? What kind of evidence will convince them?<br><br>What are the influential research institutions saying on sepsis? What language are they using?	Supply evidence on sepsis	Implement policy engagement activities on sepsis	Organise conference or conference tracks on sepsis
iv. Media	How does the media engage in sepsis?	Disseminate evidence on sepsis	Implement public engagement activities on sepsis	Commemorate World Sepsis Day
